# KLF4 functions as an activator of the androgen receptor through reciprocal feedback

**DOI:** 10.1038/oncsis.2016.79

**Published:** 2016-12-19

**Authors:** M-K Siu, F Suau, W-Y Chen, Y-C Tsai, H-Y Tsai, H-L Yeh, Y-N Liu

**Affiliations:** 1 Program for Cancer Biology and Drug Discovery, College of Medical Science and Technology, Taipei Medical University and Academia Sinica, Taipei, Taiwan; 2 Graduate Institute of Cancer Biology and Drug Discovery, College of Medical Science and Technology, Taipei Medical University, Taipei, Taiwan; 3 Department of Anesthesiology, Wan Fang Hospital, Taipei Medical University, Taipei, Taiwan; 4 Department of Microbiology, Faculty of Pharmacy, Dicle University, Diyarbakir, Turkey; 5 Department of Pathology, Wan Fang Hospital, Taipei Medical University, Taipei, Taiwan; 6 Department of Pathology, School of Medicine, College of Medicine, Taipei Medical University, Taipei, Taiwan; 7 Institute of Information System and Applications, National Tsing Hua University, Hsinchu, Taiwan

## Abstract

In prostate cancer, Krüppel-like factor 4 (KLF4) depletion occurs frequently, suggesting a role as suppressor tumor. KLF4 is a transcription factor associated with androgen receptor (AR) expression; however, its cellular functions and signaling regulation mechanism remain largely unknown. In this study, we demonstrated that activated AR binds to the *KLF4* promoter and enhances KLF4 expression, which reciprocally targets the *AR* promoter, thus sustaining KLF4 activity. Ectopic KLF4 expression in androgen-independent prostate cancer cells induced AR expression and decreased cell proliferation, invasion and bone metastasis. We previously showed that increased microRNA (miR)-1 expression is associated with reduced bone metastasis of prostate cancer cells. Here we observed that KLF4 targets the primary *miR-1-2* stem-loop promoter and stimulates miR-1 expression. In clinical prostate cancer specimens, KLF4 levels were positively correlated with miR-1 and AR levels. These data suggest that the loss of KLF4 expression is one mechanistic link between aggressive prostate cancer progression and low canonical AR output through miR-1 inactivation.

## Introductinon

Kruppel-like factor 4 (KLF4), which belongs to the KLF family of transcription factors, regulates diverse cellular functions depending on tissue and tumor type or cancer stage and may have a role as a tumor suppressor or oncogene.^1^ KLF4 inhibits tumor progression in prostate cancer and that the loss of KLF4 expression is a diagnostic marker in patients with advanced prostate cancer,^2, 3^ consistent with the role of KLF4 in cell cycle arrest and growth inhibition.^1, 4, 5^ Moreover, the loss of one allele of KLF4 can induce intestinal adenoma development in Apc^Min^ mice, which corroborates the tumor-suppressive effect of this protein.^6^ Notably, KLF4 expression is significantly correlated with activated androgen receptor (AR) signaling in prostatic stromal cells^7^ and circulating tumor cells of prostate cancer patients,^8^ suggesting an association between KLF4 and AR in prostate cancer cells.

AR is activated after the binding of androgenic ligands and has a pivotal role in prostate cancer development.^9, 10^ AR has become the most crucial therapeutic target in prostate cancer treatment.^11, 12, 13^ However, many treatments targeting AR signaling are noncurative and associated with a poor prognosis, despite continuous hormonal manipulation. Therefore, the resistance mechanisms and molecular pathways involved in changes of AR function as prostate cancer progresses are being increasingly investigated.

The microRNAs (miRs) represent a class of small, regulatory, noncoding RNA molecules that can become dysregulated in cancer.^14^ Altered miR expression and the subsequent effects on target gene transcription have been frequently associated with metastatic trait acquisition.^15^ miR-1 has been proposed as a candidate tumor suppressor and prognostic marker for prostate cancer.^16, 17, 18^ We previously established that ectopic miR-1 expression negatively affects the growth ability of prostate cancer cells.^19^ Notably, we demonstrated that miR-1 expression suppresses experimental bone metastasis and that miR-1 is directly and positively modulated by AR signaling.^20^

In this study, we explored the regulatory mechanisms linking AR signaling with miR-1 expression in prostate cancer. We demonstrated that *KLF4* expression is directly and transcriptionally upregulated by AR, and AR expression is reciprocally upregulated by KLF4. Moreover, in prostate cancer cell lines, KLF4 binds to the *miR-1* promoter and induces its expression. Our findings also revealed that KLF4 is positively associated with AR and miR-1 levels in patient tissue samples. Here, we established a central role for KLF4 in tumor suppression by connecting its regulation by AR and modulation of miR-1 expression.

## Results

### Induction of KLF4 expression is associated with activated AR signaling

**Figure 1:**
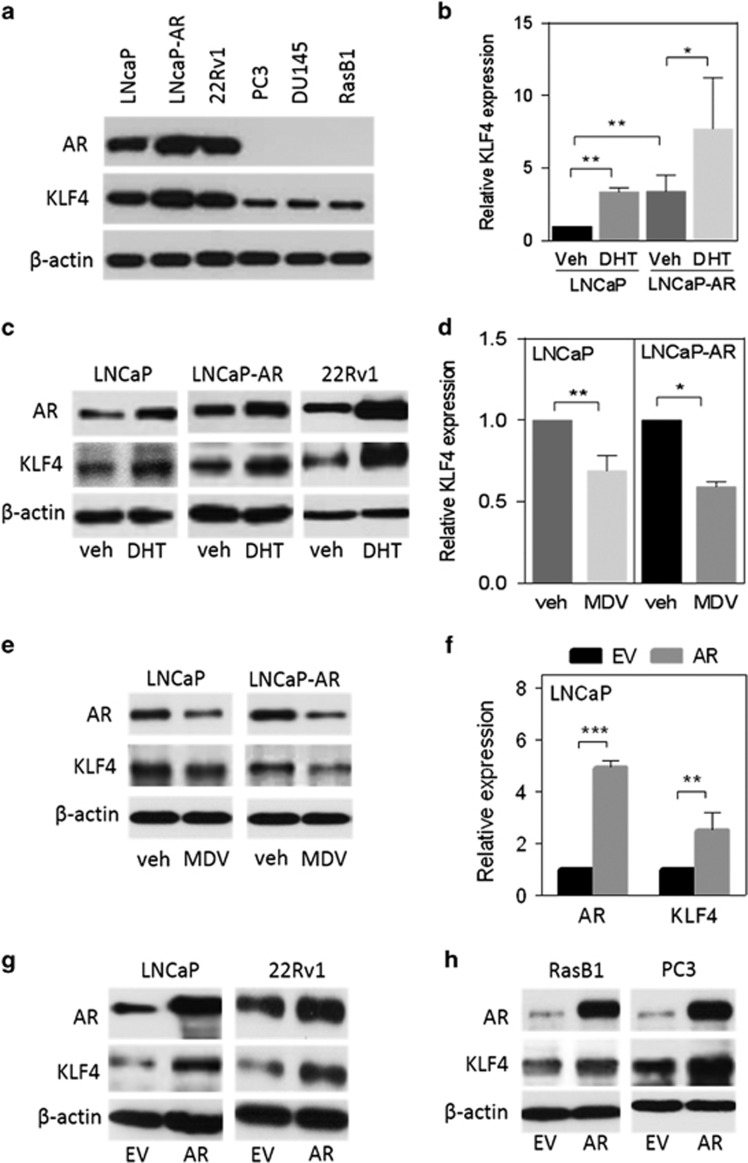
Activated androgen receptor (AR) signaling induces Kruppel-like factor 4 (KLF4) expression. (**a**) Immunoblots for detecting endogenous AR and KLF4 levels in various prostate cancer cell lines. (**b**) KLF4 mRNA levels in LNCaP and LNCaP-AR cells following DHT treatment (10 nm for 24 h in a 10% charcoal-stripped FBS-containing medium). (**c**) Immunoblots for detecting AR and KLF4 levels in LNCaP, LNCaP-AR and 22Rv1 cells following DHT treatment. (**d**) KLF4 mRNA levels in LNCaP and LNCaP-AR cells following MDV3100 treatment (10 μm for 24 h in a 10% FBS-containing medium). (**e**) Immunoblots for detecting AR and KLF4 levels in LNCaP and LNCaP-AR cells following MDV3100 treatment. (**f**) Quantitative real-time reverse-transcription PCR analysis of AR and KLF4 in LNCaP cells after ectopic AR or empty vector (EV) expression. (**g**) Representative immunoblot analysis of AR and KLF4 protein expression in LNCaP and 22Rv1 cells following EV or AR expression. (**h**) Immunoblots for detecting AR and KLF4 protein expression in RasB1 and PC3 cells following EV or AR expression. All the experiments were performed in triplicate, and the data are presented as the mean±s.e.m., *n*=3. *P<0.05, **P<0.01, ***P<0.001.

Although KLF4 activity is repressed in several cancers and can have a tumor-suppressive effect,^2, 5, 21^ the specific role of KLF4 in AR pathway-activated prostate cancer remains unclear. We analyzed the relationship between KLF4 and AR signaling and observed that AR-expressing cells LNCaP, LNCaP-AR and 22Rv1 had higher KLF4 expression than did cells without AR expression PC3, DU145 and RasB1 (Figure 1a). In LNCaP and LNCaP-AR cells, KLF4 messenger RNA (mRNA) and protein levels increased after dihydrotestosterone (DHT) treatment (Figures 1b and c), but decreased after treatment with MDV3100, an AR antagonist^22, 23^ (Figures 1d and e), suggesting an association between KLF4 and AR signaling in the same cell signaling pathway in AR-positive prostate cancer cells. Moreover, KLF4 mRNA levels increased in LNCaP cells transfected with an AR expression vector (Figure 1f). The increase in KLF4 levels was confirmed by immunoblotting extracts from LNCaP and 22Rv1 cells, demonstrating that KLF4 expression increases in the presence of AR expression (Figure 1g). These results were further confirmed by immunoblotting extracts from AR-negative RasB1 and PC3 cells ectopically overexpressing AR (Figure 1h). Taken together, these data suggest that KLF4 expression is upregulated by AR in prostate cancer.

### AR binding to the *KLF4* promoter directly and positively regulates KLF4 expression

**Figure 2:**
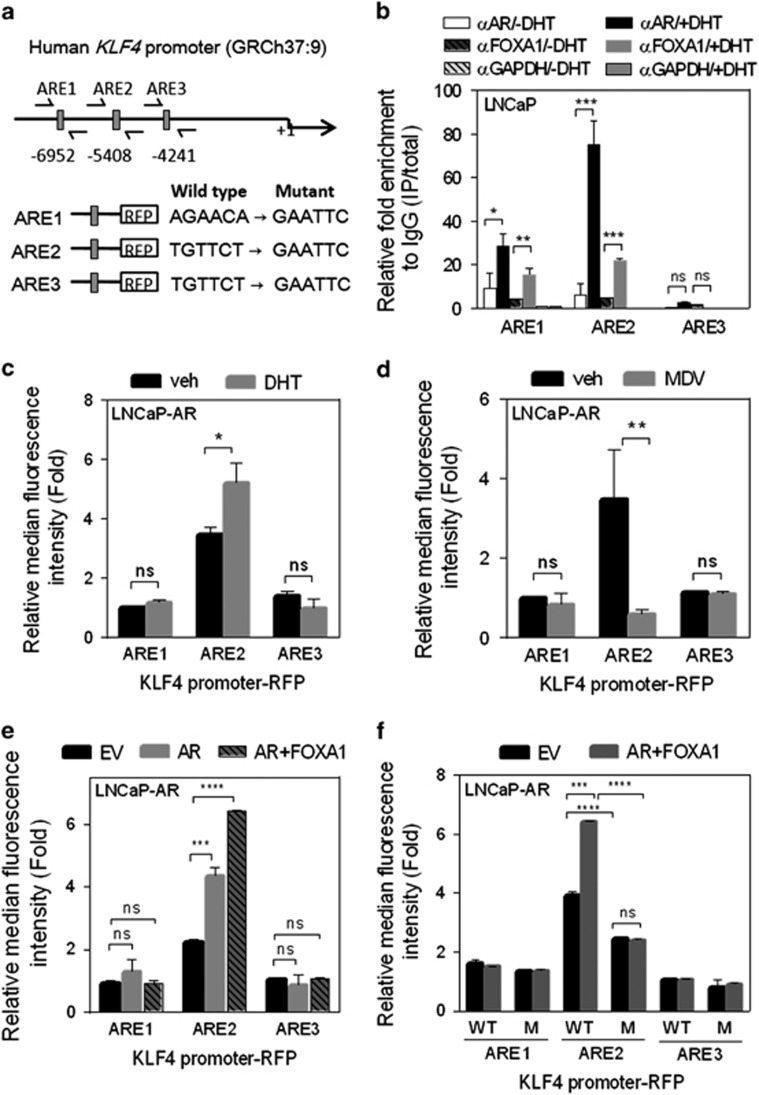
Activated androgen receptor (AR) stimulates Kruppel-like factor 4 (KLF4) expression by enhancing the *KLF4* promoter. (**a**) Schematic of the predicted AR responsive elements (AREs) and an introduced binding site mutant in promoter reporter constructs of the human *KLF4* promoter at −6952, −5408 and −4241 bp upstream. (**b**) Chromatin immunoprecipitation assays for AR and FOXA1 binding to predicted AREs in the *KLF4* promoter region measured in LNCaP cells treated with 10 nm DHT for 4 h. The binding activity of each protein to each site is given as a percentage of the total input, normalized to each IgG. (**c** and **d**) Activity of a RFP reporter gene containing AREs from the *KLF4* promoter. Expression of the transiently transfected reporter gene, normalized to gene expression from a transfected control vector, was assayed in LNCaP-AR cells following 24 h treatment with 10 nm DHT (**c**) or 10 μm MDV3100 (**d**). Relative median fluorescence intensity (MFI) of the reporter protein following DHT or MDV3100 treatment. (**e**) Activity of the same RFP reporter gene as in **c** when LNCaP-AR cells were transfected with a plasmid expressing AR, FOXA1, or a control empty vector (EV). The MFI normalized to a control for transfection efficiency is shown. (**f**) The LNCaP-AR cells were transiently cotransfected with wild-type (WT) or ARE-mutated ARE-RFP reporters with AR or FOXA1 expression vectors for 48 h. The MFI was measured through fluorescence-activated cell sorting and normalized to the value of the EV. All the experiments were performed in triplicate and the data are presented as the mean±s.e.m., *n*=3. NS, nonsignificant, *P<0.05, **P<0.01, ***P<0.001, ****P<0.0001.

We next investigated whether AR stimulates KLF4 expression through binding and transcriptional regulation of the *KLF4* promoter. We evaluated the *KLF4* promoter for the presence of potential AR-binding sites and identified three putative AR homologous responsive elements (AREs) in the upstream region of the *KLF4* promoter (Figure 2a). FOXA1, an AR cofactor, can cooperate with AR to mediate gene expression in prostate cancer.^24, 25, 26^ To determine whether AR and FOXA1 effectively bind to the *KLF4* promoter, we performed quantitative reverse-transcription PCR (qRT–PCR) analysis in LNCaP cells after a chromatin immunoprecipitation (ChIP) assay by using anti-AR and anti-FOXA1 antibodies. The binding of AR and FOXA1 was observed only at the ARE1 and ARE2 motifs, and the binding significantly increased after DHT treatment (Figure 2b). Next, to assess the effect of AR on the transcriptional activity of *KLF4*, the LNCaP-AR cells were transfected with RFP reporter constructs containing the individual predicted AREs of the *KLF4* promoter. DHT treatment of LNCaP-AR cells significantly increased ARE2 reporter activity but not that of ARE1 or ARE3 (Figure 2c); in addition, we noted concordantly decreased reporter activity after treatment of the same cells with MDV3100 (Figure 2d). Furthermore, ARE2 reporter activity increased in LNCaP-AR cells transfected with AR compared with cells transfected with a control vector, and the activity increased further in cells overexpressing both AR and FOXA1 (Figure 2e). To characterize the specificity of AR binding to the *KLF4* promoter region, we introduced point mutations in the ARE regions (Figure 2a); we observed that mutations in ARE2 disrupted the ability of AR and FOXA1 to stimulate *KLF4* promoter activity in reporter assays (Figure 2f). These data are consistent with the mechanism that AR activates KLF4 transcription through direct physical interaction with the *KLF4* promoter.

### KLF4 induces AR expression by reciprocally interacting with the *AR* promoter

**Figure 3:**
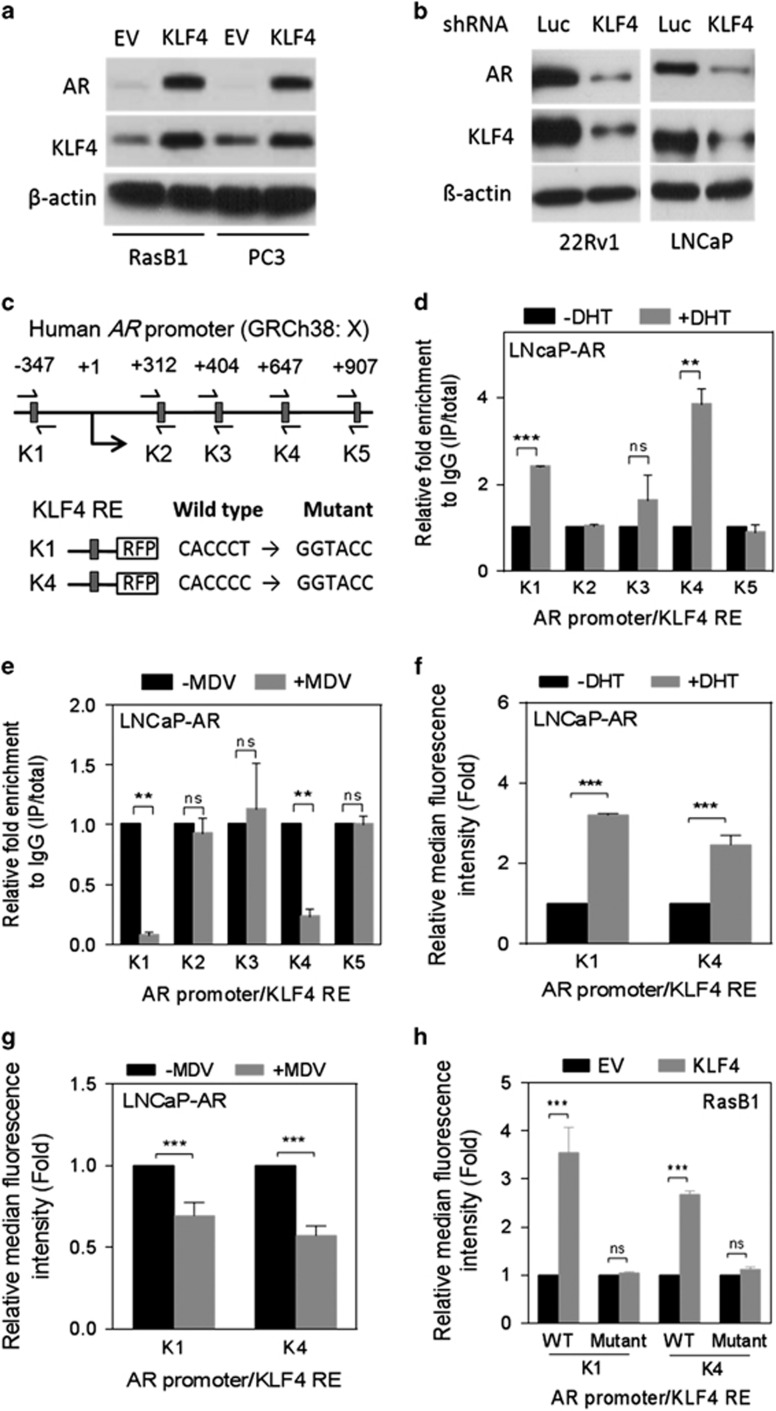
Kruppel-like factor 4 (KLF4) directly binds to the androgen receptor (*AR*) promoter and regulates its transcription activity. (**a**) Immunoblots for detecting AR and KLF4 protein expression in RasB1 and PC3 cells following KLF4 or control empty vector (EV) expression. (**b**) Immunoblots for detecting AR and KLF4 levels in 22Rv1 and LNCaP cells following KLF4 or luciferase (Luc) shRNA introduction. (**c**) Schematic of predicted KLF4-responsive elements (REs) and an introduced binding site mutant in promoter reporter constructs of the human *AR* promoter at −347 bp upstream and +312, +404, +647 and +907 bp downstream. (**d** and **e**) Chromatin immunoprecipitation assay for KLF4 binding to predicted KLF4 REs in the *AR* promoter region measured in LNCaP-AR cells treated with 10 nm DHT (**d**) and 10 μm MDV3100 (**e**) for 4 h. The binding activity of each protein to each site is given as a percentage of the total input normalized to each IgG. (**f** and **g**) Activity of an RFP reporter gene containing KLF4 REs (K1 and K4) from the *AR* promoter. Expression of the transiently transfected reporter gene, normalized to gene expression from a transfected control vector, was assayed in LNCaP-AR cells following 24 h treatment with 10 nm DHT (**f**) or 10 μm MDV3100 (**g**). Relative median fluorescence intensity (MFI) of the reporter protein following DHT or MDV3100 treatment. (**h**) The RasB1 cells were transiently cotransfected with wild-type (WT) or KLF4 RE-mutated K1- and K4-RFP reporters with KLF4 expression vectors for 48 h. The MFI was measured through fluorescence-activated cell sorting and normalized to the value of the EV. All the experiments were performed in triplicate and the data are presented as the mean±s.e.m., *n*=3. NS, nonsignificant, **P<0.01, ***P<0.001.

To determine whether KLF4 can regulate AR expression, thereby creating a regulatory loop between AR and KLF4, we analyzed the consequences of modulating KLF4 expression on AR expression in prostate cancer cells. AR mRNA levels increased in RasB1 and PC3 cells transfected with an ectopic KLF4 expression vector (Supplementary Figure S1A). Consistently, the AR and KLF4 protein levels also increased in RasB1 and PC3 cells transfected with the ectopic KLF4 expression vector (Figure 3a). Notably, the AR and KLF4 mRNA levels decreased in 22Rv1 and LNCaP cells transfected with small interfering RNA (siRNA) against KLF4 (siKLF4; Supplementary Figure S1B). Moreover, the AR and KLF4 protein levels decreased in cells transfected with a KLF4 shRNA knockdown vector (shKLF4; Figure 3b). These data strongly support the presence of positive feedback between AR and KLF4 in prostate cancer cells.

To further investigate this mechanism and determine whether KLF4 regulates AR expression at the transcriptional level, we analyzed the *AR* promoter for the presence of KLF4 homologous binding sites and identified five putative sites (Figure 3c). To assess the ability of KLF4 to bind to the *AR* promoter, we performed ChIP assays. KLF4/*AR* chromatin complexes were immunoprecipitated using an anti-KLF4 antibody from nuclear extracts of LNCaP-AR cells after DHT treatment. By using qRT–PCR, we then analyzed the KLF4-responsive element (RE) region of *AR*. We noted significantly increased nuclear KLF4-binding signals at putative K1 and K4 sites in DHT-treated cells compared with those in untreated cells (Figure 3d). By contrast, decreased nuclear KLF4-binding signals were observed at the same sites after MDV3100 treatment (Figure 3e). To determine whether the KLF4-binding sites of the *AR* promoter (K1 and K4) are functional, we conducted reporter assays by using a construct with a KLF4-RE at the K1 and K4 sites incorporated into an RFP reporter. The reporter activity increased in response to DHT (Figure 3f) and decreased after MDV3100 treatment (Figure 3g). The introduction of mutations in the KLF4 binding sites in the *AR* promoter abolished the effects of KLF4 on *AR* transcription (Figure 3h and Supplementary Figure S1C). We additionally treated AR-positive LNCaP and 22Rv1 cells with siKLF4 and observed that the *AR* promoter reporter activity decreased in the presence of siKLF4 (Supplementary Figure S1D). These data demonstrate that KLF4 directly binds to the *AR* promoter and regulates *AR* expression.

### KLF4 inhibits cell growth and motility by upregulating AR expression

**Figure 4:**
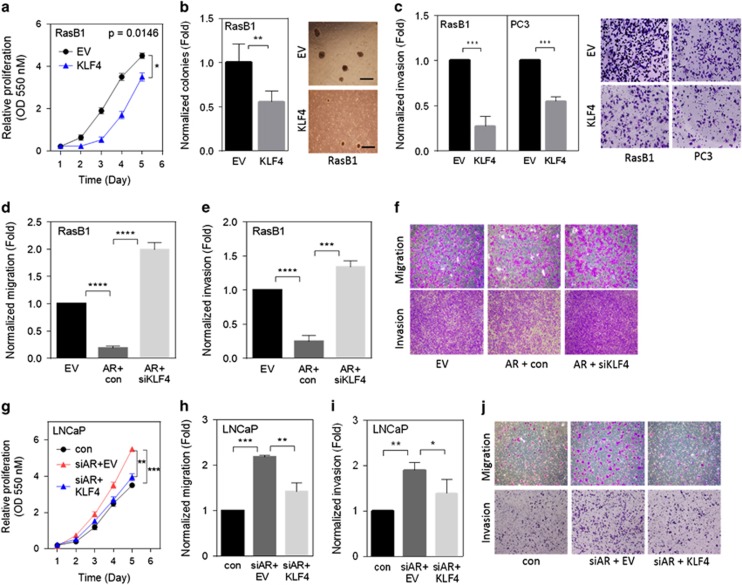
Androgen receptor (AR)-regulated Kruppel-like factor 4 (KLF4) reduced the malignant phenotypes of prostate cancer cells. (**a**) Proliferation of RasB1 cells stably overexpressing KLF4 or an empty vector (EV). All experiments were performed in triplicate and the data are presented as the mean±s.e.m., *n*=6. (**b**) Normalized colony-formation assay for RasB1 cells expressing KLF4 or the EV (left). Normalized colonies were counted with 10 microscopic images at × 100 magnification after 14 days (right). All the experiments were performed in triplicate and the data are presented as the mean±s.e.m. Scale bar, 100 μm. (**c**) Normalized invasion of RasB1 and PC3 cells stably transfected with KLF4 or EV (left). The cells were plated in a serum-free medium above Matrigel transwell filters, with an attractant in the lower well. At 16 h, the cells that had migrated through the filter to invade the lower well were quantified in five medium-power fields (right). All the experiments were performed in triplicate and the data are presented as the mean±s.e.m., *n*=3. (**d** and **e**) Normalized migration (**d**) and invasion (**e**) of RasB1 cells expressing AR or the EV following KLF4 or scramble control (con) SMARTpool siRNA expression. All the experiments were performed in triplicate and the data are presented as the mean±s.e.m., *n*=3. ***P<0.001, ****P<0.0001. (**f**) Images of migration (**d**) and invasion (**e**). (**g**) Proliferation of LNCaP cells transfected with siAR or control siRNA following KLF4 or EV expression. All the experiments were performed in triplicate and the data are presented as the mean±s.e. of the mean, *n*=6. (**h** and **i**) Normalized migration (**h**) and invasion (**i**) of LNCaP cells expressing siAR or control siRNA following KLF4 or EV expression. All the experiments were performed in triplicate and the data are presented as the mean±s.e.m., *n*=3. *P<0.05, **P<0.01, ***P<0.001. (**j**) Images of migration (**h**) and invasion (**i**).

To assess the role of KLF4 in human prostate cancer progression, we stably expressed KLF4 in RasB1 and PC3 cells (as confirmed by immunoblots in Figure 3a). KLF4 overexpression significantly reduced the growth rate of these cells *in vitro* (Figure 4a and Supplementary Figure S2A). Notably, when we expressed KLF4 in RasB1 cells, the colony formation of these cells decreased in three-dimensional growth assays in soft agar (Figure 4b). Moreover, when we treated AR-positive LNCaP and 22Rv1 cells with shKLF4 (knockdown effect as confirmed by immunoblots in Figure 3b), the colony formation of these cells increased (Supplementary Figure S2B). We further examined the functional relevance of KLF4-mediated reduction in the migration and invasion of RasB1 and PC3 cells. The cells expressing KLF4 had reduced cell motility compared with cells carrying the control vector, according to migration (Supplementary Figure S2C) and invasion (Figure 4c) assays. Moreover, the motility of the AR-positive LNCaP-AR cells through transwells was significantly induced when siKLF4 was expressed (Supplementary Figure S2D). To further confirm that cell motility was reduced by the AR-dependent induction of KLF4 expression, RasB1 and PC3 cells were stably transfected with AR and subsequently transfected with siKLF4. The cells overexpressing AR had decreased invasiveness and migration compared with cells expressing the empty vector, whereas cell invasion and migration were induced when the AR-expressing cells were treated with siKLF4 (Figures 4d–f and Supplementary Figures S2E–G). We next performed AR knockdown combined with KLF4 overexpression or AR overexpression combined with KLF4 knockdown and analyzed the effects on proliferation and motility of LNCaP and 22Rv1 cells. Remarkably, AR knockdown in AR-positive LNCaP cells induced cell proliferation, migration and invasion, whereas KLF4 overexpression in AR-knockdown cells decreased cell proliferation, migration and invasion (Figures 4g–j). Moreover, significant cell proliferation and motility were induced in AR-positive 22Rv1 cells when siKLF4 was expressed, whereas AR overexpression in KLF4-knockdown cells decreased cell proliferation, migration and invasion (Supplementary Figures S2H–K). These results demonstrate that KLF4 and AR can compensate each other in the negative regulation of proliferation, migration and invasion.

**Figure 5:**
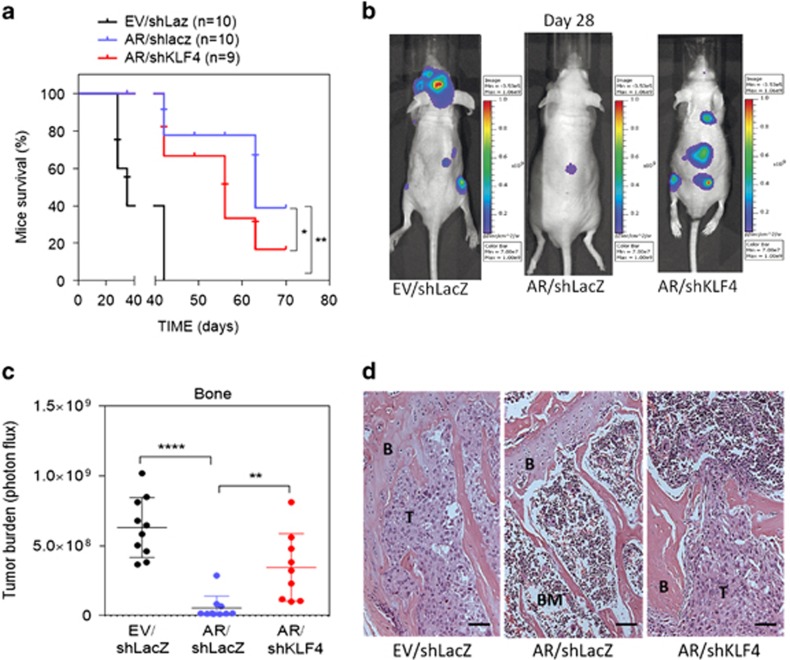
Reduced Kruppel-like factor 4 (KLF4) expression increased bone metastasis in association with androgen receptor (AR) expression. (**a**) Survival rates of tumor-bearing mice with RasB1/empty vector (EV) or RasB1/AR cells expressing a control shLacZ or KLF4 shRNA vector (RasB1/EV/shLacZ, *n*=10; RasB1/AR/shLacZ, *n*=10; RasB1/AR/shKLF4, *n*=9). (**b**) Representative bioluminescence imaging (BLI) in tumor-bearing mice at day 28 after injection with the same cells as in **a**. (**c**) Quantification of the BLI signals of the mice with bone metastases in **b**. (**d**) Representative histological images of bone metastases in tumor-bearing mice inoculated with the same cells as in **b**. B, bone; BM, bone marrow; T, tumor. Scale bar, 100 μm. Log-rank test was used for survival curve analysis. *P<0.05, **P<0.01, ****P<0.0001.

These results were further supported by *in vivo* experiments. We intracardially administered AR-overexpressing RasB1 cells to mice and noted an increase in survival rates (Figure 5a) and a considerable decrease in bone metastasis (Figure 5b). However, the administration of AR-expressing cells with shKLF4 significantly increased bone metastasis and decreased survival rates compared with mice injected with control shRNA-harboring cells (Figures 5a–d), confirming that the AR-dependent induction of KLF4 expression is associated with bone metastasis. In summary, the proliferation and metastatic abilities of prostate cancer cells are closely regulated by KLF4 expression, supporting its role as a tumor suppressor in prostate cancer.

### KLF4 induces miR-1 expression by directly binding to primary *miR-1* promoter

**Figure 6:**
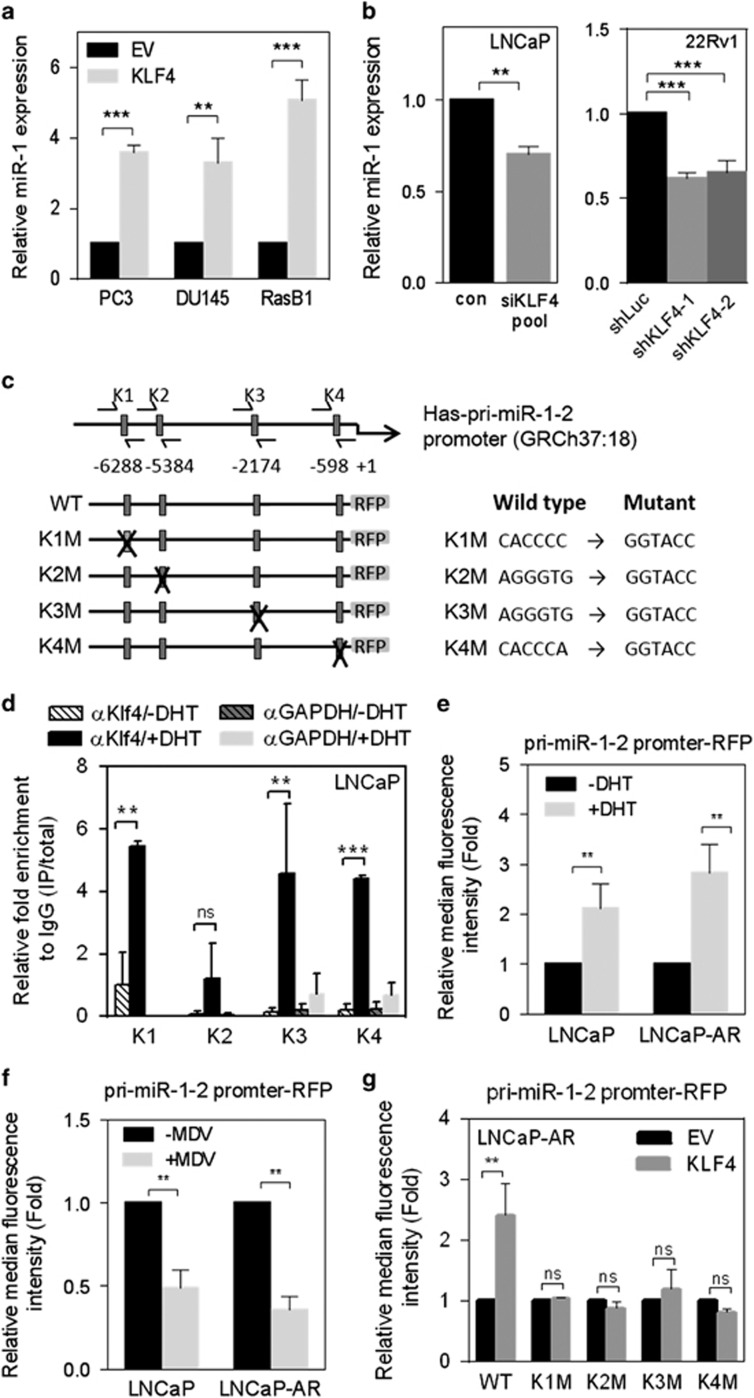
Kruppel-like factor 4 (KLF4) transcriptionally upregulates microRNA (miR)-1 by binding to the *pri*-*miR-1-2* stem-loop promoter. (**a**) Quantitative reverse-transcription PCR (qRT–PCR) analysis of miR-1 in LNCaP, LNCaP-AR and RasB1 cells expressing KLF4 or an empty vector (EV) for 48 h. (**b**) qRT–PCR analysis of miR-1 in LNCaP or 22Rv1 cells following the introduction of a control or KLF4 SMARTpool siRNA (left) or a control luciferase (Luc) or KLF4 shRNA vector (right) for 48 h. (**c**) Schematic of the predicted KLF4-binding sites (K1 to K4) and an introduced binding site mutant in the human *pri*-*miR-1-2* stem-loop promoter at −6288, −5384, −2174 and −598 bp upstream. (**d**) Chromatin immunoprecipitation assays for KLF4 binding to predicted KLF4 binding sites measured in LNCaP cells treated with 10 nm DHT for 4 h. The binding activity of each protein to each site is given as a percentage of the total input normalized to each IgG. (**e** and **f**) LNCaP and LNCaP-AR cells were transiently transfected with a *pri-miR-1-2* stem-loop promoter reporter following 48 h treatment with 10 nm DHT (**e**) or 10 μm MDV3100 (**f**). The relative median fluorescence intensity (MFI) of the reporter protein following DHT or MDV3100 treatment is shown. (**g**) The LNCaP-AR cells were transiently cotransfected with wild-type (WT) or mutated KLF4 binding site (K1M to K4M) reporters with KLF4 or an EV for 48 h. The MFI was measured through fluorescence-activated cell sorting and normalized to the value of the EV. All the experiments were performed in triplicate and the data are presented as the mean±s.e.m., *n*=3. NS, nonsignificant, **P<0.01, ***P<0.001.

To investigate the tumor-suppressor role of KLF4, we focused on identifying KLF4 target substrates potentially involved in tumorigenesis prevention. We previously showed that activated AR can directly target miR-1 and that miR-1 functions as an inhibitor of prostate cancer bone metastasis.^20^ Accordingly, we analyzed the relationships between the genes associated with high miR-1 expression and androgen-responsive gene signatures^27^ in the Taylor Prostate Cancer Dataset.^28^ By using a bioinformatics approach, GSEA, we determined an association between KLF4 expression and AR-induced miR-1 expression (Supplementary Figures S3A,B). Next, we determined whether miR-1 levels are associated with KLF4 expression in prostate cancer. First, we observed that KLF4 overexpression in AR-negative prostate cancer cell lines increased miR-1 expression (Figure 6a). KLF4 inhibition through the transient transfection with siKLF4 in LNCaP or stable transfection with shKLF4 in 22Rv1 prostate cancer cells reduced endogenous miR-1 levels (Figure 6b). Thus, we hypothesized that KLF4 stimulates miR-1 expression by interacting with the primary *miR-1* stem-loop promoter. We then analyzed the primary *miR-1* stem-loop promoter for a homologous RE of KLF4 and observed four candidate binding elements for KLF4 in the upstream promoter region of the primary *miR-1-2* gene (that is, *pri-miR-1-2*; Figure 6c). To identify which of these sites is required to stimulate miR-1 expression, we performed ChIP assays in LNCaP cells left untreated or treated with DHT by using an anti-KLF4 or control anti-GAPDH antibody. qRT–PCR analyses indicated a significant increase in KLF4-binding signals at the K1, K3 and K4 sites after DHT treatment (Figure 6d). We then performed reporter assays in prostate cancer cell lines by using the wild-type *pri-miR-1-2* stem-loop promoter, containing KLF4-REs driving RFP reporter expression. Compared with untreated cells, reporter gene activity significantly increased in DHT-treated LNCaP and LNCaP-AR cells (Figure 6e), whereas it decreased in MDV3100-treated cells (Figure 6f). Moreover, KLF4 overexpression increased wild-type *pri-miR-1-2* reporter activity; however, a single mutation in each of the four KLF4 binding sites could disrupt the ability of KLF4 to induce *pri-miR-1-2* reporter activity (Figure 6g). These results indicate that KLF4 regulates miR-1 expression through the direct binding of KLF4 to the *pri-miR-1-2* promoter region.

In a previous report, we demonstrated that AR can promote *pri-miR-1-2* transcription.^20^ Here, to study the contribution of KLF4 and AR in *pri-miR-1-2* transcription, we first determined miR-1 levels in AR-positive cells after knocking down AR, KLF4 or both. Our qRT–PCR results indicated that the simultaneous knockdown of siAR and siKLF4 resulted in the highest downregulation of miR-1 expression (Supplementary Figure S3C, left). We also examined miR-1 levels in AR-negative cells after overexpressing AR and/or KLF4. Cotransfection with AR and KLF4 induced the highest miR-1 levels (Supplementary Figure S3C, right). Moreover, the cotransfection of AR-positive LNCaP cells with the *pri-miR-1-2* promoter reporter and siAR, siKLF4 or both siRNA induced the lowest reporter activity (Supplementary Figure S3D, left), whereas overexpression of both the KLF4 and AR expression vectors in AR-negative RasB1 cells induced the highest reporter activity (Supplementary Figure S3D, right). These results suggest that AR and KLF4 cooperatively promote the transcription of *pri-miR-1-2*.

### KLF4 expression is positively correlated with AR and miR-1 levels in clinical prostate cancer samples

**Figure 7:**
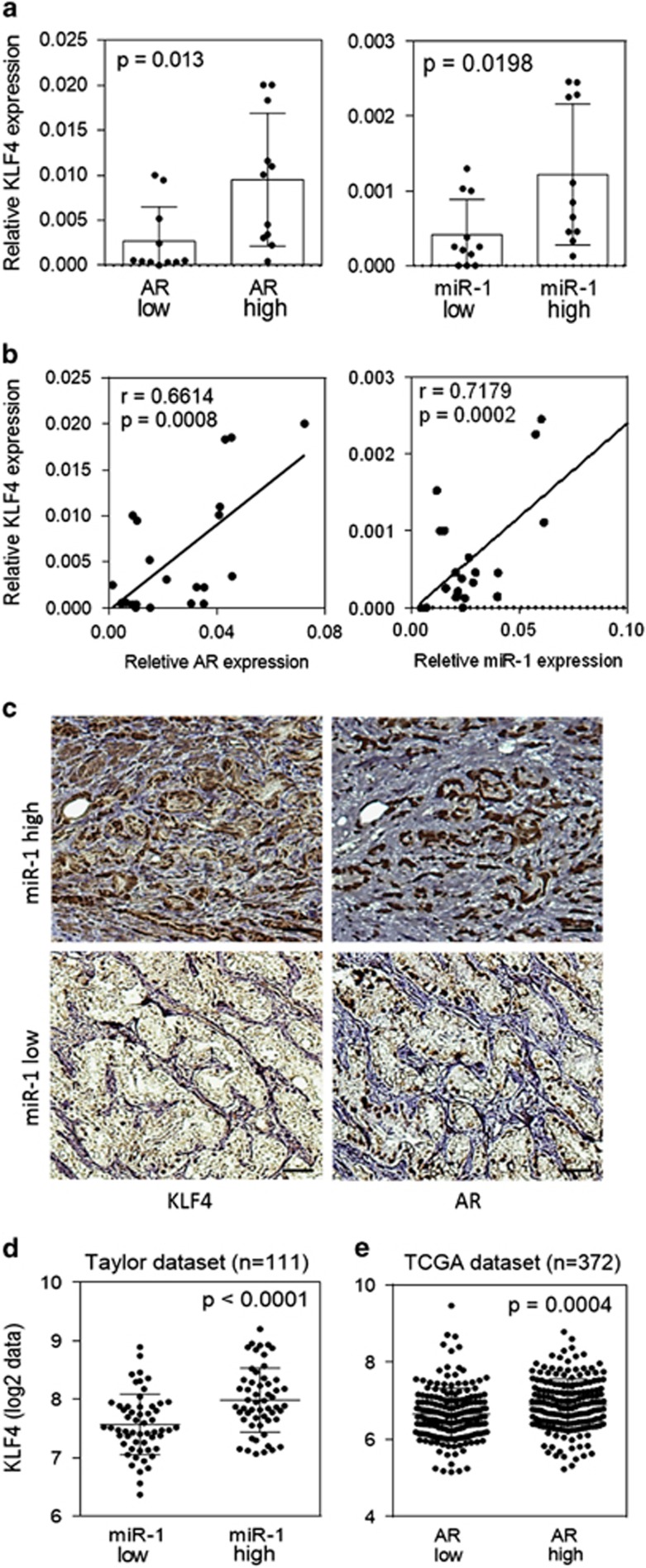
Increased Kruppel-like factor 4 (KLF4) expression is positively correlated with androgen receptor (AR) and microRNA (miR)-1 expression in prostate cancer patients. (**a**) KLF4 levels in two tissue sample groups divided on the basis of AR and miR-1 levels (*n*=11 per group). (**b**) Pearson correlation coefficient analysis of the association between the mean KLF4 and mean AR and miR-1 mRNA expression levels in independent primary prostate samples (*n*=22). Significance was determined according to the Gaussian population (Pearson) and a two-tailed test. (**c**) Immunohistochemistry with KLF4- and AR-specific antibodies in prostate cancer tissue sections with different miR-1 levels. Scale bars represent 100 μm. (**d**) KLF4 levels in two tissue sample groups containing 98 primary tumor and 13 distant metastasis samples separated on the basis of miR-1 expression from the Taylor Prostate Cancer Dataset. Significance was determined using the Student's *t*-test. (**e**) KLF4 levels in two tissue sample groups containing 372 primary tumor samples separated on the basis of AR expression from the The Cancer Genome Atlas (TCGA) data set. Significance was determined using the Student's *t*-test.

To further study the link between KLF4 and its targets AR and miR-1 in clinical samples, we analyzed 22 independent prostate tumor tissue samples collected from the Wan Fang Hospital, Taipei Medical University (Taiwan). The samples were divided into two groups of low and high levels of both AR and miR-1 expression, as measured using qRT–PCR. Analysis of variance confirmed that KLF4 expression levels were significantly higher in the groups with high AR or miR-1 expression levels (Figure 7a). Similarly, KLF4 expression was positively correlated with AR and miR-1 expression according to a Pearson coefficient correction analysis (Figure 7b). Through immunohistochemistry, we observed that tumor tissues with high miR-1 expression levels exhibited strong cytoplasmic and nuclear staining for KLF4 (Figure 7c—top, left). Moreover, strong nuclear staining for AR was observed in the same tissue samples (Figure 7c—top, right). Notably, the expression of both KLF4 and AR was low in tumor tissue samples with low miR-1 expression (Figure 7c—bottom).

We next validated the relationship between AR, KLF4 and miR-1 expression in human prostate cancer tissues from the Taylor Prostate Cancer Dataset and The Cancer Genome Atlas. The analysis of variance results indicated that KLF4 expression was induced in tissue samples with high miR-1 and AR levels (Figures 7d and e). We also noted that KLF4 and miR-1 levels were negatively associated with cancer grading (Supplementary Figures S4A,B). To confirm the positive correlation between KLF4 and AR signaling in prostate cancer, clinical samples from the Taylor Prostate Cancer Dataset were divided into two groups with either high or low KLF4 expression, based on a measure of the relative mRNA expression using z-scores. A z-score analysis of the mean showed higher KLF4 expression in samples with upregulated androgen-dependent genes^27^ (Supplementary Figure S4C). Furthermore, GSEA using two androgen-responsive gene sets^24, 27^ showed a positive association between high KLF4 expression and androgen-responsive gene sets in clinical samples from the Taylor Prostate Cancer Dataset (Supplementary Figures S4D,E). Therefore, prostate cancer samples with increased AR levels had increased KLF4 expression. Taken together, our findings identify a regulatory mechanism by which AR upregulates *KLF4* expression directly and transcriptionally; subsequently, KLF4 increases miR-1 expression levels and sustains suppressed prostate tumorigenesis. In addition, *AR* expression is reciprocally upregulated by KLF4, creating an amplifying retropositive loop.

## Discussion

KLF family proteins are transcription factors involved in the regulation of several cellular processes, including proliferation, apoptosis, differentiation, inflammation, migration and tumor formation.^1^ Our studies have focused on the role of KLF4 in prostate carcinogenesis, particularly in metastasis inhibition. AR is a member of the family of intracellular steroid hormone receptors and functions as a ligand-dependent transcription factor.^29^ Here, we identified a self-reinforcing regulatory loop for the KLF4-AR axis that involves the AR-induced transcriptional stimulation of the *KLF4* promoter, indicating that *KLF4* is a candidate target gene for AR and *AR* is a candidate target gene for KLF4. In concordance with the tumor-suppressive effect mediated by KLF4 activation, we demonstrated that AR-induced KLF4 expression negatively regulates the proliferation and metastatic abilities of prostate cancer cells. We further showed that KLF4 increases miR-1 expression levels and sustains as a tumor suppressor of prostate cancer. KLF4 inhibition in several cancer types, including prostate cancer, may contribute to cellular hyperproliferation and malignant transformation.^4, 5, 21, 30, 31^ Our previous report showed that KLF4 is a direct transcriptional inhibitor of SLUG expression in prostate cancer cells,^2^ suggesting a role of KLF4 in inhibiting cell migration and invasion in prostate cancer. Regarding the cell cycle, KLF4 can cause either G1 or G2 arrest in different cell types.^32, 33, 34^ Studies have indicated that KLF4 functions as a tumor suppressor in regulating cell proliferation and apoptosis on the basis of the modulation of downstream genes, such as those of p27,^30^ p21,^35^ p57,^36^ cyclin D1 (ref. 37) and cyclin B1,^38^ and that *KLF4* has potent tumor suppressor-like function. miR-1 has been described as a suppressor of prostate cancer.^16, 17, 19^ We previously reported the loss of AR-mediated activation of miR-1 and subsequent activation of SRC-stimulated prostate cancer bone metastasis.^20^ Although activated AR stimulates miR-1 transcription by binding to its promoter,^20^ our present results support a model where AR might function as a transcription factor that activates the KLF4-miR-1 signaling pathway to persist the tumor-suppressive role of miR-1. This finding is consistent with our results showing reduced KLF4 and miR-1 levels were negatively associated with elevated cancer grading.

A combination of androgen-deprivation therapy and surgical prostatectomy or radioablation has been used to treat therapy-naive patients with prostate cancer.^39, 40^ Although the initial response is typically efficient, almost all patients develop castration-resistant prostate cancer, frequently leading to patient death.^41^ Our current study provides a novel explanation of this phenomenon: androgen-deprivation therapy results in AR signaling inhibition, leading to KLF4 and miR-1 repression and potentially contributing to castration-resistant prostate cancer. Alternatively, AR inhibition may cause the inactivation of KLF4-repressing tumorigenesis pathway components that affect the biological functions of cells. Several lines of evidence have suggested that during prostate cancer development, AR has a dual function as an oncogene^42^ and a tumor suppressor.^43^ Moreover, the transcription profiles of AR could be very different between androgen-dependent and -independent prostate cancers.^24^ Our current study supported the tumor suppressor role of AR by indicating an AR-KLF4 positive feedback loop, which can inhibit malignant phenotypes in AR-negative or androgen-insensitive prostate cancer cells (PC3 and RasB1). We also studied the underlying mechanisms contributing to the resistance to androgen-deprivation therapy and poor AR antagonist efficacy in advanced prostate cancer. Our results may aid in understanding individual intervariability in drug responses to consequently improve drug therapies: by modulating KLF4 expression to repress cancer malignancy, an alternative target may be identified to improve the efficacy of current prostate cancer therapy modalities.

In conclusion, *KLF4* expression is directly and transcriptionally upregulated by AR, and *AR* expression is reciprocally upregulated by KLF4. This study highlights the role of the KLF4-AR axis in androgen deprivation and reveals pivotal mechanisms responsible for castration-resistant prostate cancer development. Despite the gaps in the understanding of KLF4 and its interplay with cell motility and proliferation pathways, we present a novel function for the KLF4-miR-1 axis—a new putative predictive and surveillance biomarker of antiandrogen therapy for advanced prostate cancer.

## Materials and methods

### Cells, reagents and constructs

Androgen-dependent LNCaP-AR (parental LNCaP overexpressing wild-type AR) and metastatic RasB1 (DU145 expressing a constitutively active Ras) cell lines were obtained from Dr Kathleen Kelly (NCI/NIH, Bethesda, MD, USA) and maintained as described previously.^20, 44, 45, 46, 47, 48^ DU145, PC3, LNCaP and 22Rv1 cell lines were from the American Type Culture collection (Manassas, VA, USA). All the cells were cultured in the RPMI 1640 medium supplemented with 10% FBS (fetal bovine serum). After DHT treatment (10 nm; Sigma, St Louis, MO, USA), cells were cultured for 24 h in a 10% charcoal-stripped FBS-containing medium. Some cells were treated with the AR antagonist MDV3100 (Selleck, Houston, TX, USA) at 10 μm for 24 h in a 10% FBS-containing medium. The cells with stable or transient AR, FOXA1, or KLF4 expression were established through transfection with AR, FOXA1 or KLF4 expression vectors, respectively; an empty vector, pCDH-CMV-MCS-EF1-Puro (System Biosciences, Palo Alto, CA, USA) with a puromycin-selectable marker, was used as a control. The siRNA (scramble and siKLF4) and shRNA vectors (shLuc and shKLF4) were purchased from Thermo Scientific (Dharmacon SMARTpool siRNA Reagents; Waltham, MA, USA) and the RNAi Core Lab (Academia Sinica, Taipei, Taiwan). Transient transfection of the plasmids and siRNAs was performed using the X-tremeGENE HP DNA transfection reagent (Roche, Clovis, CA, USA) and Lipofectamine RNAiMAX (Invitrogen, Waltham, MA, USA), respectively. By using GRCh37, KLF4 binding sites were located on human *pri-miR-1-2* and *AR* on chromosomes 18 and X, respectively, and AR binding sites were located on human *KLF4* on chromosome 9 (Supplementary Table S1). The *AR*, *KLF4* and *pri-miR-1-2* promoters with AR- and KLF4-binding site-RFP reporter vectors were constructed using the Clone-it Enzyme Free Lentivectors Kit (System Biosciences). All the primers used for these constructs are listed in Supplementary Table S2. All the constructs were verified using DNA sequence analysis.

### Western blot analysis

The cells grown on six-well plates (10^6^ cells/well) were lysed in 150 μl of RIPA buffer containing complete protease inhibitors (Roche) and phosphatase inhibitors (Roche), 25 mm β-glycerophosphate, 10 mm sodium fluoride and 1 mm sodium vanadate. Twenty micrograms of protein was separated per lane through SDS-gel electrophoresis. After transfer to polyvinylidene fluoride membrane, the blots were blocked with 5% bovine serum albumin in phosphate-buffered saline plus Tween 20. Primary antibodies were incubated overnight at 4 °C, and secondary antibodies were incubated at room temperature for 1 h as indicated in Supplementary Table S3.

### qRT–PCR analysis

We measured KLF4, AR and miR-1 expression in the human prostate cancer cell lines, with or without DHT or MDV3100 treatment and AR or KLF4 overexpression, by using qRT–PCR. Total RNA was isolated using the mirVana PARIS RNA isolation system (Ambion, Waltham, MA, USA). For reverse transcription, 3 μg of total RNA was used with the SuperScript III kit (Invitrogen). In the amplification step, SYBR green PCR master mix (Applied Biosystems, Waltham, MA, USA) was used. For all primer pairs, the thermocycler was run for an initial 95 °C incubation for 10 min, followed by 40 cycles with 95 °C for 15 s and 60 °C for 1 min. All the reactions were normalized to human *GAPDH* and run in triplicate. All the primers used for PCR are listed in Supplementary Table S4. miR-1 RT-PCRs were performed using the TaqMan MicroRNA Assays kit (Applied Biosystems). All the values were normalized to a human *SNORD48* endogenous control and run in triplicate.

### ChIP assay

The cells were treated with or without DHT (10 nm) for 4 h. The cultured cells (10^7^) were cross-linked with 1% formaldehyde at room temperature for 15 min. Fixation was quenched with glycine, and the cells were washed twice with cold phosphate-buffered saline containing a complete protease inhibitor (Roche). The cell pellets were resuspended in cell lysis buffer and incubated on ice for 15 min. Nuclei were collected by centrifugation at 10^4^ r.p.m. and 4 °C for 10 min and resuspended in nuclear lysis buffer. Chromatin was sheared using a sonicator (Branson Sonifier 250, Dietzenbach, Germany) with a microtip in a 20 s burst followed by 1 min of cooling on ice for a total sonication time of 5 min per sample. This procedure results in DNA fragment sizes of approximately 100 to 300 bp. Sheared chromatin was divided to perform immunoprecipitation with a rabbit IgG antibody (Santa Cruz Biotechnology, Dallas, TX, USA) or primary antibody at 4 °C overnight. Immunoprecipitation, washing, elution, reverse cross-linking and DNA purification steps were performed according to Millipore's protocol. A qRT–PCR was performed in triplicate with 2 μl of eluted chromatin. ChIP antibodies and PCR primers are listed in Supplementary Table S5. Predictions for transcription factor-binding sites within promoter regions were adopted from the AliBaba 2.1 program (gene-regulation.com).

### Promoter reporter assay

Promoter function was analyzed through FACS (fluorescence-activated cell sorting), and relative median fluorescent intensity was measured from the first peak of fluorescence, as described previously.^20, 47, 48^ The cells were treated with or without 10 nm DHT and 10 μm MDV3100 for 48 h. The median fluorescent intensity for RFP was measured through FACS by using FACSDiva software and normalized to the value of the vehicle. Three independent experiments were performed in triplicate.

### Proliferation assay

The PC3 and RasB1 cells were transfected with the KLF4 expression vector at a density of 2000 cells/well. Each day, the cells were stained with 0.5% crystal violet fixative solution for 15 min, washed with distilled water and allowed to air dry. At the end of the experiment, crystal violet was dissolved by adding 100 μl of 50% ethanol containing 0.1 m sodium citrate to each well, and absorbance was measured at 550 nm on an ELISA reader (Biocompare, South San Francisco, CA, USA).

### Colony-formation assay

Colony-formation assays were performed using a starting cell count of 5 × 10^4^ cells/well. Single-cell suspensions of KLF4-transfected RasB1 cells or shKLF4 vector-transfected LNCaP-AR or DU145 cells were seeded in six-well plates in the RPMI 1640 medium supplemented with 10% FBS. The colonies were counted on day 21 after plating in triplicate and normalized with a control shLuc vector.

### Invasion and migration assays

For invasion and migration assays, KLF4 or AR expression vector-transfected PC3 and RasB1 cells with a control siRNA or siKLF4 or siKLF4-transfected LNCaP-AR cells were resuspended at a concentration of 2.5 × 10^5^ cells/ml in a serum-free medium. We purchased Matrigel from BD Biosciences (San Jose, CA, USA) for the invasion assay. Matrigel-coated transwell dishes were prepared by adding 200 μl of Matrigel diluted 10-fold with the serum-free medium. The cells were plated at 2.5 × 10^5^ per well in the serum-free medium above the Matrigel. The lower chamber was filled with 600 μl of the serum-containing medium. The cells that had invaded the Matrigel-coated transwells after 12 h were fixed and stained with a 0.5% crystal violet fixative solution for 15 min. The invading cells on the underside of the membrane were counted and quantified in five medium-power fields for each replicate in triplicate. In the migration assay, we used transwells without Matrigel, and the cells were fixed and stained as described for the invasion assay.

### Metastasis and survival assays in mice

Animal experiments were performed according to the protocol approved by the Taipei Medical University Animal Care and Use Committee (Taiwan). To analyze metastasis, 7-week-old male nude mice (NLAC, Taipei, Taiwan) were intracardially administered either 10^5^ RasB1 human metastasis prostate cancer cells harboring a luciferase expression vector with a control empty or AR expression vector or 10^5^ AR-transfected RasB1 cells with a control shLacZ or shKLF4 expression vector. Bioluminescence imaging was performed double blinding at 28 days after injection as described previously.^46^ In all cases, the same mice were followed for up to 10 weeks after injection to measure the accumulation of bone metastases through bioluminescence imaging as well as the survival percentage. For survival studies, mice were killed upon 10% body weight loss, paralysis or head tilting.

### Immunohistochemistry

We collected 22 independent primary prostate tumor samples from the Wan Fang Hospital, Taipei Medical University (Taiwan). The study was approved by the Wan Fang Hospital, Taipei Medical University Institutional Review Board (approval no.: N201512033) and performed according to the approved guidelines. Immunohistochemistry was performed using anti-KLF4 (Sigma) and anti-AR (Epitomics, Burlingame, CA, USA) antibodies at 1:500 and 1:250 dilutions, respectively, as described in Supplementary Materials and Methods.

### Statistical analysis

All the data are presented as the mean±s.e.m. Statistical calculations were performed on GraphPad Prism analysis tools (GraphPad Software, La Jolla, CA, USA). Differences between individual groups were determined using the Student's *t*-test or one-way analysis of variance, followed by Bonferroni's post test for comparisons among three or more groups. The method for determining the cutoff points was predecided by considering half the number of patients. A *P*-value of <0.05 was considered statistically significant.
