# Supplementary material for: KLF4 functions as an activator of the androgen receptor through reciprocal feedback
Source: Oncogenesis. 2016 Dec 19;5(12):e282–. doi: 10.1038/oncsis.2016.79 (PMC5177777; doi:10.1038/oncsis.2016.79)
Supplement: Supplementary Information [file oncsis201679x1.docx]

**KLF4 functions as an activator of androgen receptor through a reciprocal feedback**

Man Kit Siu, Florent Suau, Wei-Yu Chen, Yuan-Chin Tsai, Hong-Yuan Tsai, Hsiu-Lien Yeh, Yen-Nien Liu

**Supplemental Information**

**SUPPLEMENTAL MATERIALS AND METHODS**

**Immunohistochemical (IHC) Staining**

We collected 22 independent primary prostate tumors from Wan Fang Hospital, Taipei Medical University (Taiwan). The study was approved by the Taipei Medical University-Wan Fang Hospital Institutional Review Board (approval no.: N201512033) and carried out in accordance with the approved guidelines. IHC was performed using KLF4 (Sigma, HPA002926, MO, USA) and AR (Epitomics, #3184-1, CA, USA) antibPage 1odies at respective 1: 500 and 1: 250 dilutions, respectively. In general, unstained sections were deparaffinized and rehydrated. Antigen retrieval was performed using the Target Antigen Retrieval Solution (DAKO, CA, USA) and autoclaved for 10 min. Endogenous peroxidase was blocked using a 3% hydrogen peroxide solution. All sections were blocked with Cyto Q Background Buster Reagent (Innovex BioSciences, CA, USA). Primary antibodies were incubated overnight at 4 °C in Antibody Diluent with Background Reducing Components (DAKO). A secondary antibody, 1:250 horseradish peroxidase (HRP)-labeled anti- mouse/rabbit (Vector Laboratories, CA, USA), was incubated at room temperature for 30 min, and bound peroxidase was detected using the ABC Peroxidase Kit (Vector Laboratories) and DAB (DAKO). All IHC slides were counterstained with hematoxylin. For histomorphometric analysis of tissue sections, microscopic images were examined under 200x magnification using an Axioplan microscopy system (Zeiss, NY, USA).

**Clinical Outcomes and Correlation Analyses Using Human Datasets**

We used miRs and mRNA expression data from the Taylor prostate cancer dataset ^1^ and the Cancer Genome Atlas (TCGA) prostate cancer dataset. The study using the Taylor dataset was accessed from the Memorial-Sloan Kettering Cancer Center (MSKCC) Cancer Genomics data portal (http://cbio.mskcc.org/cancergenomics/prostate/data/) on 7/27/2012, and clinical and publicly available gene expression and microRNA expression data on 98 primary and 13 metastatic prostate cancer samples were downloaded. The study using TCGA dataset was selected Level 3 normalized microarray gene expression data (UNC_AgilentG4502A_07) from TCGA database (https://tcga-data.nci.nih.gov/tcga/; 11/21/2014) of 372 primary prostate tumors from patients treated by a radical prostatectomy. Expression data (and resulting z-scores) were log2-normalized. Gene set enrichment analysis (GSEA) software was downloaded from the Broad Institute ^2^. Androgen-responsive ^3, 4^ gene signatures were used to determine correlations with AR, KLF4, and miR-1 levels. The number of permutations was set to 1000, and the permutation type was set to “phenotype”. A normalized enrichment score (NES) and false discovery rate (FDR) were calculate by the program. Correlations of miR-1 and KLF4 with the gene set were suggested by NES values. For the z-score analysis, gene sets were scored by summing the expression z-scores per tumor within the cohort. Tumors were mean-stratified by their AR, miR-1, and KLF4 expressions, and the mean expression of each of these genes was determined in each group.

**Supplemental Figure**

**Supplementary Figure S1.** Kruppel-like factor 4 (KLF4) is positively associated with androgen receptor (AR) expression in prostate cancer cells. (A) Quantitative reverse-transcription (qRT)-PCR analysis of AR in RasB1 and PC3 cells following KLF4 or empty vector (EV) expression. (B) qRT-PCR analysis of AR and KLF4 in 22Rv1 and LNCaP cells following introduction of KLF4 or control (con) SMARTpool siRNA. (C) PC3 cells were transiently cotransfected with wild-type (WT) or KLF4 responsive element (RE)-mutated K1- and K4-GFP reporters with a KLF4 expression vector or EV for 48 h. The median fluorescence intensity (MFI) was measured through fluorescence-activated cell sorting and normalized to the value of the EV. (D) LNCaP and 22Rv1 cells were transiently cotransfected with WT K1- and K4-RFP reporters with siKLF4 or control siRNA for 48 h. The MFI was measured through fluorescence-activated cell sorting and normalized to the value of the control siRNA. All experiments were performed in triplicate and data are presented as the mean ± standard error of the mean, *n* = 3. ns: nonsignificant, * *p* < 0.05, ** *p* < 0.01, *** *p* < 0.001, **** *p* < 0.0001.

**Supplementary Figure S2.** Androgen receptor (AR)-regulated Kruppel-like factor 4 (KLF4) reduces prostate cancer cell proliferation and motility. (A) Proliferation of PC3 cells stably overexpressing KLF4 or an empty vector (EV). (B) Colony formation assay for LNCaP and 22Rv1 cells transfected with KLF4 or luciferase (Luc) shRNA (Left). Normalized colonies were counted with 10 microscopic images at 100× magnification after 14 days (Right). Scale bar: 100 μm. (C) Normalized migration of RasB1 and PC3 cells stably transfected with KLF4 or EV (Left). The cells were plated in a serum-free medium above transwell filters with an attractant in the lower well. At 16 h, the cells that had migrated through the filter to invade the lower well were quantified in five medium-power fields (Right). (D) Normalized migration of LNCaP-AR cells with KLF4 or control (con) SMARTpool siRNA expression. (E and F) Normalized migration (E) and invasion (F) of PC3 cells expressing AR or the EV following siKLF4 or control siRNA expression. (G) Images of migration (E) and invasion (F). (H) Proliferation of 22Rv1 cells transfected with siKLF4 or control siRNA following AR or EV expression. (I and J) Normalized migration (I) and invasion (J) of 22Rv1 cells expressing siKLF4 or control siRNA following AR or EV expression. (K) Images of migration (I) and invasion (J). All experiments were performed in triplicate and data are presented as the mean ± standard error of the mean, *n* = 3. * *p* < 0.05, ** *p* < 0.01, *** *p* < 0.001, **** *p* < 0.0001.

**Supplementary Figure S3.** Increased Kruppel-like factor 4 (KLF4) expression is associated with activated androgen receptor (AR) signaling and cooperated with AR to promote *pri-miR-1-2* transcription. (A) GSEA of the Taylor Prostate Cancer Dataset, showing the enrichment of miR-1 expression among gene sets, the expression of which was increased in association with an androgen-responsive signature. NES, normalized enrichment score; FDR, false discovery rate. (B) GSEA from (A) showing *KLF4* as a candidate gene for an upregulated androgen-responsive signature of samples with high microRNA (miR)-1 expression from the Taylor Prostate Cancer Dataset. (C, left) Quantitative reverse-transcription (qRT)-PCR analysis of the miR-1 levels in LNCaP cells expressing siAR, siKLF4, or control siRNA for 48 h. (C, right) qRT-PCR analysis of the miR-1 levels in RasB1 cells expressing AR, KLF4, or an empty vector (EV) for 48 h. (D, left) LNCaP cells were transiently cotransfected with the *pri-miR-1-2* promoter reporter with siAR, siKLF4, or control siRNA for 48 h. (D, right) RasB1 cells were transiently cotransfected with the *pri-miR-1-2* promoter reporter with AR, KLF4, or an EV for 48 h. The MFI was measured through fluorescence-activated cell sorting and normalized to the value of the control siRNA or EV. All experiments were performed in triplicate and data are presented as the mean ± standard error of the mean, *n* = 3. ns: nonsignificant, * *p* < 0.05, ** *p* < 0.01, *** *p* < 0.001, **** *p* < 0.0001.

**Supplementary Figure S4.** Increased Kruppel-like factor 4 (KLF4) expression is associated with activated androgen receptor (AR) signaling. (A and B) KLF4 (A) and microRNA (miR)-1 (B) expression in patient samples in the Taylor Prostate Cancer Dataset by Gleason score. Significance was determined through a one-way analysis of variance. * vs. Gleason score 6. *** *p* < 0.001, **** *p* < 0.0001. (C) The AR-upregulated signature genes were expressed as summary z-scores for samples separated on the basis of KLF4 expression above or below the median value in the Taylor Prostate Cancer Dataset. (D and E) GSEAs showed the enrichment of upregulated androgen-responsive gene sets in Taylor prostate cancer tissues expressing high KLF4 levels by using the gene sets of Nelson et al. ^4^ (D) and Wang et al. ^3^ (E)

**References**

1 Taylor BS, Schultz N, Hieronymus H, Gopalan A, Xiao Y, Carver BS *et al*. Integrative genomic profiling of human prostate cancer. Cancer Cell 2010; 18: 11-22.

2 Subramanian A, Tamayo P, Mootha VK, Mukherjee S, Ebert BL, Gillette MA *et al*. Gene set enrichment analysis: a knowledge-based approach for interpreting genome-wide expression profiles. Proc Natl Acad Sci U S A 2005; 102: 15545-15550.

3 Wang Q, Li W, Zhang Y, Yuan X, Xu K, Yu J *et al*. Androgen receptor regulates a distinct transcription program in androgen-independent prostate cancer. Cell 2009; 138: 245-256.

4 Nelson PS, Clegg N, Arnold H, Ferguson C, Bonham M, White J *et al*. The program of androgen-responsive genes in neoplastic prostate epithelium. Proc Natl Acad Sci U S A 2002; 99: 11890-11895.

**Supplemental Tables**

**Supplemental Table S1.** Bindings sites located of human *KLF4* and *pri-miR-1-2*, and *AR* gene.

| Site | Position |
| --- | --- |
| *KLF4*/ARE1 | GRCh37:9:110247049 |
| *KLF4*/ARE2 | GRCh37:9:110245530 |
| *KLF4*/ARE3 | GRCh37:9:110244377 |
| *pri-miR-1-2*/K1 | GRCh37:18:19405227 |
| *pri-miR-1-2*/K2 | GRCh37:18:19404322 |
| *pri-miR-1-2/*K3 | GRCh37:18:19401113 |
| *pri-miR-1-2*/K4 | GRCh37:18:19399537 |
| *AR*/K1 | GRCh38:X:67543686 |
| *AR*/K2 | GRCh38:X:67544346 |
| *AR*/K3 | GRCh38:X:67544438 |
| *AR*/K4 | GRCh38:X:67544681 |
| *AR*/K5 | GRCh38:X:67544941 |

**Supplemental Table S2.** Primer sequences of the promoter reporter constructs.

| Human *KLF4* promoter AR-binding elements reporter construct | |
| --- | --- |
| *KLF4*/ARE1 P1 | GCAAGTACCTTTTCTGGCCA |
| *KLF4*/ARE1 P2 | TGCCCCTTCTTTCATTTCTGA |
| *KLF4*/ARE1 P3 | gaggcagcagagaccgGCAAGTACCTTTTCTGGCCA |
| *KLF4*/ARE1 P4 | cgaacagagagagaccgTGCCCCTTCTTTCATTTCTGA |
| *KLF4*/ARE1M F | GAAAACAGTGCTAAGACTGTTCTTGATAGCTGT |
| *KLF4*/ARE1M R | TGTGTACTATACGACACTTTTGTCACGATTCT |
| *KLF4*/ARE2 P1 | GCAAGTACCTTTTCTGGCCA |
| *KLF4*/ARE2 P2 | TGCCCCTTCTTTCATTTCTGA |
| *KLF4*/ARE2 P3 | gaggcagcagagaccgGCAAGTACCTTTTCTGGCCA |
| *KLF4*/ARE2 P4 | cgaacagagagagaccgTGCCCCTTCTTTCATTTCTGA |
| *KLF4*/ARE2M F | GAAAACAGTGCTAAGACTGTTCTTGATAGCTGT |
| *KLF4*/ARE2M R | TGTGTACTATACGACACTTTTGTCACGATTCT |
| *KLF4*/ARE3 P1 | GCAAGTACCTTTTCTGGCCA |
| *KLF4*/ARE3 P2 | TGCCCCTTCTTTCATTTCTGA |
| *KLF4*/ARE3 P3 | gaggcagcagagaccgGCAAGTACCTTTTCTGGCCA |
| *KLF4*/ARE3 P4 | cgaacagagagagaccgTGCCCCTTCTTTCATTTCTGA |
| *KLF4*/ARE3M F | GAAAACAGTGCTAAGACTGTTCTTGATAGCTGT |
| *KLF4*/ARE3M R | TGTGTACTATACGACACTTTTGTCACGATTCT |
| Primary *hsa-mir-1*-2 promoter KLF4-binding elements reporter construct | |
| *pri-mir-1-2*/K1 P1 | ACCAGGGAATCATTCTAGACTCT |
| *pri-mir-1-2*/K1 P2 | TCAGATTTGTGTGGGGTGGT |
| *pri-mir-1-2*/K1 P3 | gaggcagcagagaccgACCAGGGAATCATTCTAGACTCT |
| *pri-mir-1-2*/K1 P4 | cgaacagagagagaccgTCAGATTTGTGTGGGGTGGT |
| *pri-mir-1-2*/K1M F | CTCTATTCTCTATAGGTACCCACACAAATCT |
| *pri-mir-1-2*/K1M R | GGTCAGAGGAAAGGTGAGATAAGAGATATG |
| *pri-mir-1-2*/K2 P1 | ACACTTAAGGAGCGGGAAGT |
| *pri-mir-1-2*/K2 P2 | CTGGGCCCAAAGATGACCT |
| *pri-mir-1-2*/K2 P3 | gaggcagcagagaccgACACTTAAGGAGCGGGAAGT |
| *pri-mir-1-2*/K2 P4 | cgaacagagagagaccgCTGGGCCCAAAGATGACCT |
| *pri-mir-1-2*/K2M F | AGTTCACCTCCTTGAGGTACCTACATAAATT |
| *pri-mir-1-2*/K2M R | TCCTCGCCCTTCAATATCAAGTGGAGGAACT |
| *pri-mir-1-2*/K3 P1 | TCCTAAGAAGTCAGAAGAACCCA |
| *pri-mir-1-2*/K3 P2 | ACAGAACCCAAAACACACCT |
| *pri-mir-1-2*/K3 P3 | gaggcagcagagaccgTCCTAAGAAGTCAGAAGAACCCA |
| *pri-mir-1-2*/K3 P4 | cgaacagagagagaccgACAGAACCCAAAACACACCT |
| *pri-mir-1-2*/K3M F | GGGCAAAGAAAAACAGGTACCATGTTTACAG |
| *pri-mir-1-2*/K3M R | GGTTACATAAGACCTCCCGTTTCTTTTTGT |
| *pri-mir-1-2*/K4 P1 | GCATCAAAGTGCAAACTTCAGG |
| *pri-mir-1-2*/K4 P2 | AAACCAGGATGAGTGTGGGT |
| *pri-mir-1-2*/K4 P3 | gaggcagcagagaccgGCATCAAAGTGCAAACTTCAGG |
| *pri-mir-1-2*/K4 P4 | cgaacagagagagaccgAAACCAGGATGAGTGTGGGT |
| *pri-mir-1-2*/K4M F | TTAAAAAAGTTCAAGGTACCCACACTCATCC |
| *pri-mir-1-2*/K4M R | TTTTTAAGTATTAGAAATTTTTTCAAGTTAA |
| Human *AR* promoter KLF4-binding elements reporter construct | |
| *AR*/K1 P1 | TCCTGAAAAGAACCCCTGGC |
| *AR*/K1 P2 | AGGCCAGCACTCACCAAATT |
| *AR*/K1 P3 | gaggcagcagagaccgTCCTGAAAAGAACCCCTGGC |
| *AR*/K1 P4 | cgaacagagagagaccgAGGCCAGCACTCACCAAATT |
| *AR*/K1M F | CCCTCCCTCGCCTCGGTACCGTTGGTTTTTT |
| *AR*/K1M R | GGATAGCAGGAAAAGGAGGGAGGGAGCGGAG |
| *AR*/K4 P1 | TTCCCCCACTCTCTCTCCAC |
| *AR*/K4 P2 | ACTGAAGACCTGACTGCCTTT |
| *AR*/K4 P3 | gaggcagcagagaccgTTCCCCCACTCTCTCTCCAC |
| *AR*/K4 P4 | cgaacagagagagaccg ACTGAAGACCTGACTGCCTTT |
| *AR*/K4M F | CTCCTGCCTTCCCGGTACCGAGTGCGGAGCC |
| *AR*/K4M R | GGTGAGAGAGAGGTGGAGGAGGACGGAAGGG |

**Supplemental Table S3.** Antibody information for Western blotting.

| Primary antibody | Clonality | Source | Dilution | Secondary antibody | Source | Dilution |
| --- | --- | --- | --- | --- | --- | --- |
| AR | Rabbit  monoclonal | Epitomics (#3184-1) | 1/1000 | anti-rabbit IgG | Jackson Labs | 1/5000 |
| KLF4 | Rabbit polyclonal | Cell Signaling (#4038) | 1/1000 | anti-rabbit IgG | Jackson Labs | 1/5000 |
| GAPDH | Mouse monoclonal | Novus (NB300-221) | 1/1000 | anti-mouse IgG | Jackson Labs | 1/5000 |
| β-actin | Rabbit polyclonal | GeneTex (GTX109639) | 1/1000 | anti-rabbit IgG | Jackson Labs | 1/20000 |

**Supplemental Table S4.** Primer sequences for the qRT-PCR.

| Gene | 5'-3' |
| --- | --- |
| KLF4 F | CTCCCATCTTTCTCCACGTT |
| KLF4 R | GAAGTCGCTTCATGTGGGA |
| AR F | TCTTGTCGTCTTCGGAAATG |
| AR F | TCTGGGTTGTCTCCTCAGTG |
| GAPDH F | GGACTCATGACCACAGTCCA |
| GAPDH R | CCAGTAGAGGCAGGGATGAT |

**Supplemental Table S5.** Antibody information and primer sequences for the ChIP assay.

| ChIP antibodies | | | | | |
| --- | --- | --- | --- | --- | --- |
| Primary antibody | Species | | Clonality | Source | Dilution |
| AR | Rabbit | | Polyclonal | Epitomics (#3184-1) | 1/50 |
| FOXA1 | Rabbit | | Polyclonal | Abcam (ab34814) | 1/50 |
| KLF4 | Rabbit | | Polyclonal | Abcam (ab23738) | 1/50 |
| GAPDH | Mouse | | Monoclonal | Novus (NB300-221) | 1/50 |
| IgG | Rabbit | | Polyclonal | Santa Cruz (sc-2027) | 1/50 |
| IgG | Mouse | | Polyclonal | Santa Cruz (sc-2343) | 1/50 |
| ChIP primers | | | | | |
| Site | | 5'-3' | | | |
| *KLF4*/ARE1 F | | GCAAGTACCTTTTCTGGCCA | | | |
| *KLF4*/ARE1 R | | TGCCCCTTCTTTCATTTCTGA | | | |
| *KLF4*/ARE2 F | | GCAAGTACCTTTTCTGGCCA | | | |
| *KLF4*/ARE2 R | | TGCCCCTTCTTTCATTTCTGA | | | |
| *KLF4*/ARE3 F | | GCAAGTACCTTTTCTGGCCA | | | |
| *KLF4*/ARE3 R | | TGCCCCTTCTTTCATTTCTGA | | | |
| *AR*/K1 F | | TCCTGAAAAGAACCCCTGGC | | | |
| *AR*/K1 R | | AGGCCAGCACTCACCAAATT | | | |
| *AR*/K2 F | | TGCCTTTGTCCTCCTCCTCT | | | |
| *AR*/K2 R | | GCTGAGAGTAGCCGACTGAG | | | |
| *AR*/K3 F | | CTCAGTCGGCTACTCTCAGC | | | |
| *AR*/K3 R | | CTCGCAGCCAAAGGGAGTTA | | | |
| *AR*/K4 F | | TTCCCCCACTCTCTCTCCAC | | | |
| *AR*/K4 R | | ACTGAAGACCTGACTGCCTTT | | | |
| *AR*/K5 F | | TGGGCATCTTTTGAATCTACCCT | | | |
| *AR*/K5 R | | GCCTCAAAGTCTCGTGCAGA | | | |
| *pri-mir-1-2*/K1 F | | ACCAGGGAATCATTCTAGACTCT | | | |
| *pri-mir-1-2*/K1 R | | TCAGATTTGTGTGGGGTGGT | | | |
| *pri-mir-1-2*/K2 F | | ACACTTAAGGAGCGGGAAGT | | | |
| *pri-mir-1-2*/K2 R | | CTGGGCCCAAAGATGACCT | | | |
| *pri-mir-1-2*/K3 F | | TCCTAAGAAGTCAGAAGAACCCA | | | |
| *pri-mir-1-2*/K3 R | | ACAGAACCCAAAACACACCT | | | |
| *pri-mir-1-2*/K4 F | | GCATCAAAGTGCAAACTTCAGG | | | |
| *pri-mir-1-2*/K4 R | | AAACCAGGATGAGTGTGGGT | | | |
