# Supplementary figures and images for: KLF4 functions as an activator of the androgen receptor through reciprocal feedback
Source: Oncogenesis. 2016 Dec 19;5(12):e282–. doi: 10.1038/oncsis.2016.79 (PMC5177777; doi:10.1038/oncsis.2016.79)

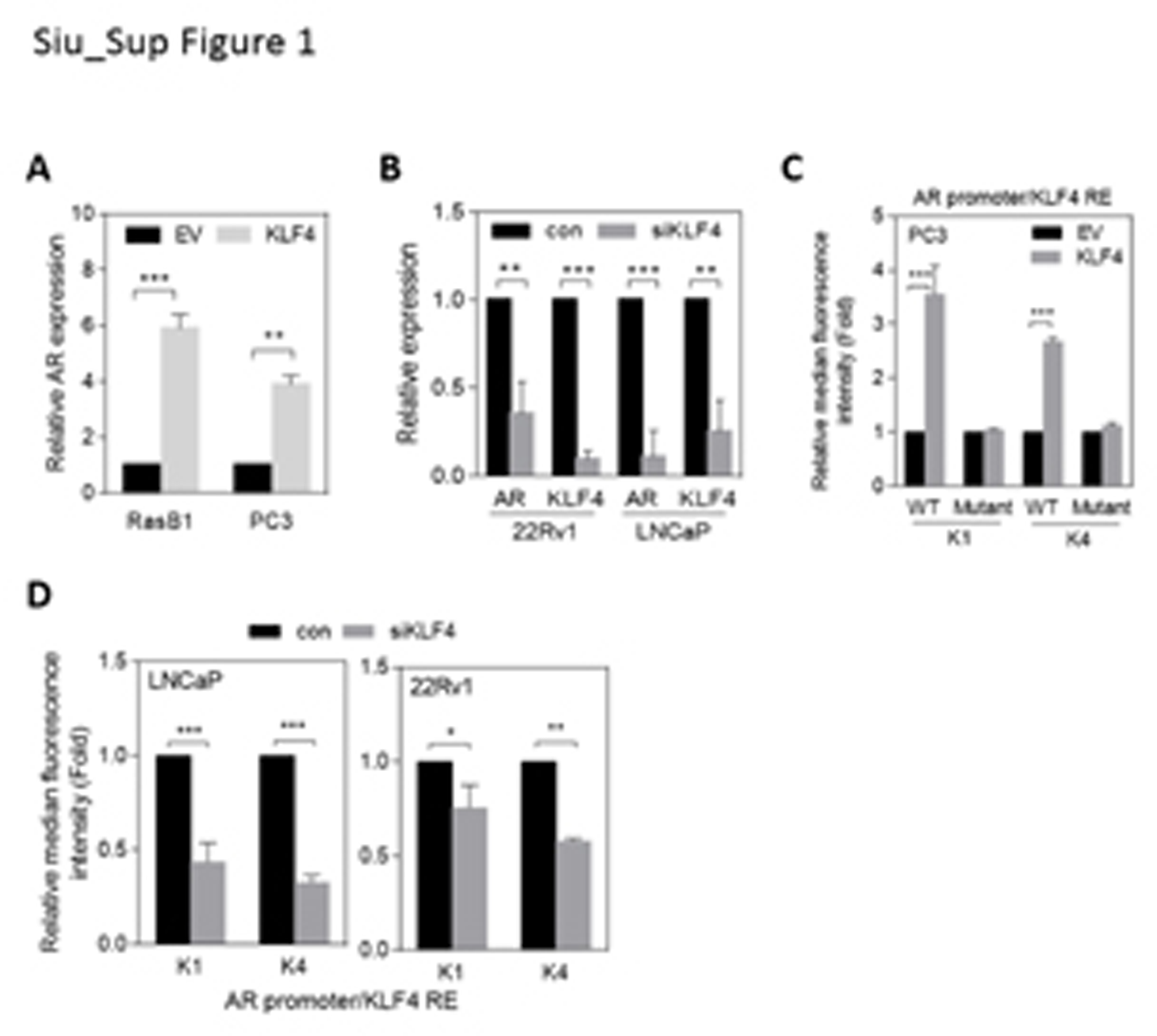

Supplement: Supplementary Figure 1 [file oncsis201679x2.tif]

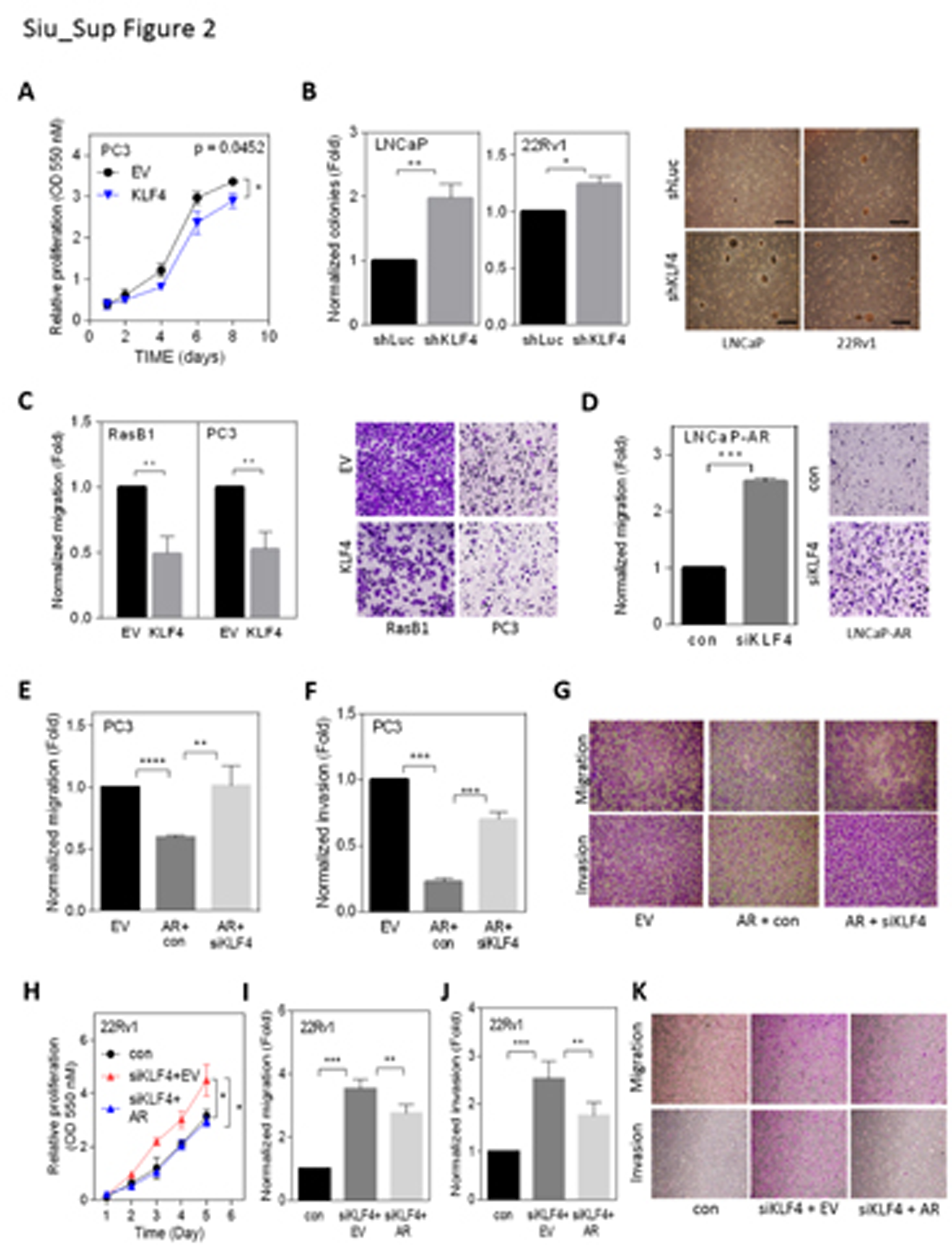

Supplement: Supplementary Figure 2 [file oncsis201679x3.tif]

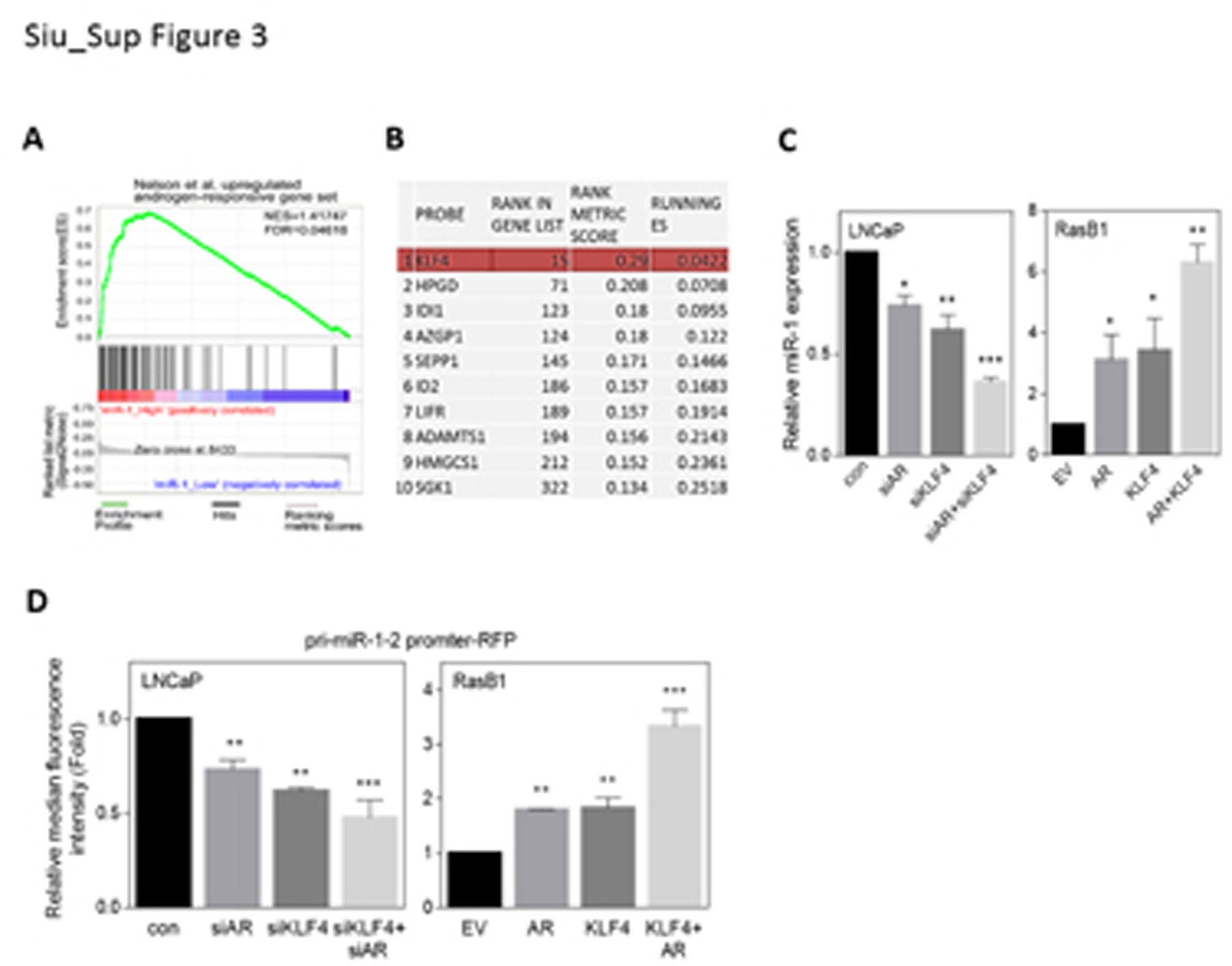

Supplement: Supplementary Figure 3 [file oncsis201679x4.tif]

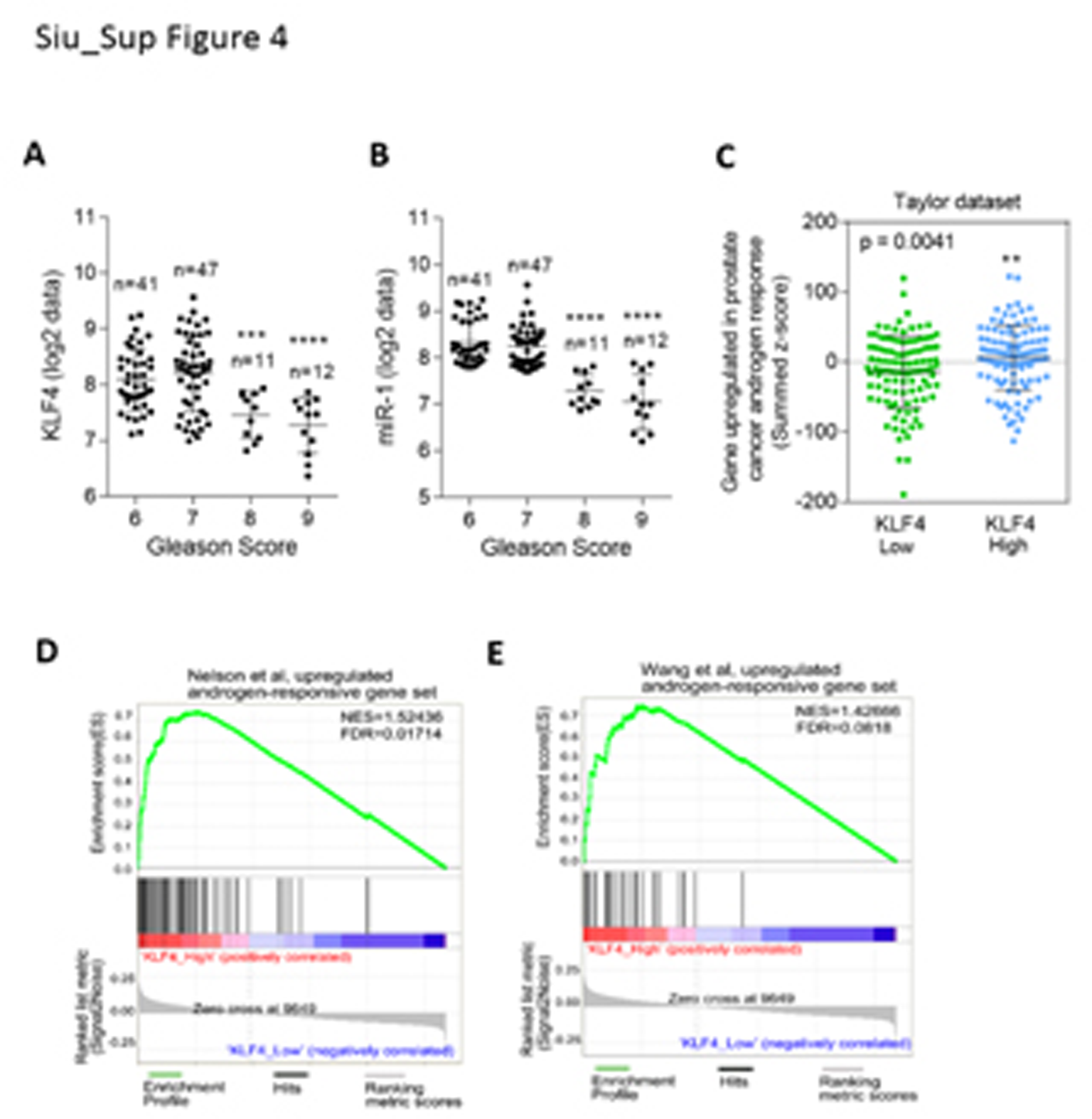

Supplement: Supplementary Figure 4 [file oncsis201679x5.tif]
